# Ten simple rules and a template for creating workflows-as-applications

**DOI:** 10.1371/journal.pcbi.1010705

**Published:** 2022-12-15

**Authors:** Michael J. Roach, N. Tessa Pierce-Ward, Radoslaw Suchecki, Vijini Mallawaarachchi, Bhavya Papudeshi, Scott A. Handley, C. Titus Brown, Nathan S. Watson-Haigh, Robert A. Edwards

**Affiliations:** 1 Flinders Accelerator for Microbiome Exploration, Flinders University, Adelaide, South Australia, Australia; 2 Department of Population Health and Reproduction, University of California, Davis, California, United States of America; 3 CSIRO Agriculture and Food, Urrbrae, South Australia, Australia; 4 Department of Pathology & Immunology, Washington University School of Medicine, St. Louis, Missouri, United States of America; 5 Alkahest Inc., San Carlos, California, United States of America; Dassault Systemes BIOVIA, UNITED STATES

This is a *PLOS Computational Biology* Software paper.

## Introduction

As bioinformatics analyses increase in size and complexity, workflow managers are becoming more popular for building pipelines [1–3]. Workflow managers, such as Snakemake [4], Nextflow [5], and Cromwell [6] with WDL or CWL [7], empower researchers to build robust pipelines that call a series of tools and scripts to perform a bespoke analysis. Workflow managers enable non-bioinformaticians to run published pipelines with confidence, and workflow managers with graphical user interfaces such as Galaxy [8] and BioWorkflow [9] have helped non-bioinformaticians create their own simple pipelines. Earlier tools for workflow management have been around for a while, including GNU Make, ruffus [10], doit [11], rake for ruby [12], and Makeflow [13]. However, the integration of cluster and cloud computing support in Snakemake, Nextflow, and Cromwell helped drive their current popularity. The use of workflow managers facilitates following the FAIR (Findable, Accessible, Interoperable, Reusable) guiding principles for open scientific research [14]. Interestingly, many existing bioinformatics command line tools are wrappers for a series of other software, but since that is the goal of workflow managers, they can be used instead. Examples of command line tools built on a workflow manager include Hecatomb [15,16], ATLAS [17], VirSorter2 [18], spacegraphcats [19], BlobToolKit [20], and PGAP [21]. These tools all consist of two key components: a *convenience launcher*, which provides the command line interface for the tool and compiles the configuration from user command line arguments, and the *workflow pipeline* and associated files, which performs the actual analysis.

Developing bioinformatics software is much quicker and easier when not reinventing the wheel. For instance, if a tool needs to parse a GenBank file, is it better to code that process manually, or simply load a library that is designed to robustly parse and validate these files? The same concept applies to how bioinformatics software runs. It is possible to write functions to compare file timestamps to add reentrancy to the software, catch error codes, and throw meaningful messages when system calls fail, validate and cleanup intermediate files, allow interaction with a job scheduling system, and perform steps in isolated containers, etc. When workflow managers were still in their infancy, the authors of several popular genome assemblers [22,23] understood the value of such functionality and invested the time to manually incorporate them into their tools. However, we now have the ability to use dedicated workflow managers to reduce the burden of developers having to include these capabilities directly within their end user software.

Writing bioinformatics software using a workflow manager is a very good idea. However, shipping the software as the native workflow script is probably a very bad idea. Workflow managers have a large, and often bewildering, number of command line arguments for controlling many aspects of how a workflow runs. Furthermore, setting up a run is usually a multistep process often requiring some or all of the following: copying the workflow repository, creating a configuration file, installing the workflow manager and dependencies, coming up with a suitable run command and scheduler interaction for your system, and executing the analysis. This can be overwhelming for users, especially those who may have no experience with the workflow manager. It is best to make using the bioinformatics tool as easy as possible in order to improve the user experience. As an added bonus, this will help maximise the user base and subsequent citations. In all of the above examples, the bioinformatics tools hide the workflow manager backend behind a simple and focused interface that is much more appealing for the end user. This simple interface helps the user to define the settings that actually matter, such as the required file inputs, instead of manually generating a configuration file with this information. Modifying the configuration file is no longer necessary but remains an option for advanced customisation. This interface also lets the user run your tool in as little as two steps: install with a package manager, run the tool.

*# Example running a workflow script*

git clone https://github.com/gituser/my_tool.git

cd my_tool

conda install --file requirements.txt

cp config/config.yaml.

nano config.yaml

mv /home/user/my_data.

snakemake --cores 16 --configfile config.yaml \

  --rerun-incomplete --printshellcmds --show-failed-logs \

  --use-conda --conda-frontend mamba

*# Example workflow as an application*

conda install my_tool

my_tool run --input /home/user/my_data --threads 16

Snakemake and Nextflow are two increasingly popular workflow management systems. They make it relatively easy to create high-quality, scalable, and reproducible bioinformatics pipelines, and they have many features that make them powerful frameworks for bioinformatics. The invitingly simple syntax makes for highly readable code and relatively quick and painless code development. The mountain of functionality they add for validating inputs, outputs, directories, reentrancy, resource management, etc. would take an exorbitant amount of time to code manually.

We present these ten simple rules for developers to follow which will facilitate their user communities in adopting and executing their workflows with fewer barriers, using examples with Snakemake and Nextflow. These rules are not intended to outline general best practices for workflow managers nor for writing command line programs. Nevertheless, you should follow any best practices outlined in the documentation for your chosen workflow manager, which will make for better, more reliable, and readable workflows. For the command line script, there are guidelines you should follow that discuss best practices for command line applications [24–26], and the resulting tool itself should be workflow ready [27].

We provide Cookiecutter [28] templates to help developers get started with adapting their own workflows for command line execution. Cookiecutter takes information about the user and their project and populates a collection of template files with this information. Our templates also double as fully functioning examples of our ten simple rules and are available for both Snakemake (github.com/beardymcjohnface/Snaketool) and Nextflow (github.com/beardymcjohnface/Nektool).

## Rule 1: Empower users to troubleshoot a workflow

No matter how much time and effort is put into making the pipeline steps robust and reliable, there will still be things that fail on different user systems, data, and use-cases. You and the end user need to be able to troubleshoot the issue as quickly and painlessly as possible. Workflow managers support defining log files for capturing standard error messages. These will help keep the terminal window clear of jargon and will save the error messages for troubleshooting. Furthermore, it is usually possible to print the location of an error log for a step that has failed, as well as display the contents of the log for failed steps. Lastly, you could write a small function to create a crash report that collects the relevant error logs and run settings information for failed steps.

Similarly, benchmarking files can be helpful for the end users, especially if they are running into steps that are taking longer than expected to finish or have to adhere to service unit requirements on high-performance computing (HPC) clusters. It is also extremely useful for test runs when trying to gauge the computational resources you will need when scaling up to larger datasets. Workflow managers are usually able to save benchmarking information for all steps to their own benchmarking files or generate run reports at the end of a run.

*# Snakemake example*

rule prinseq:

  input: inDir + "/{sample}.fq"

  output: outDir + "/prinseq/{sample}.trimmed.fq"

  **log: logDir +** "/prinseq/**{sample}.stderr**"

  **benchmark: logDir +** "/prinseq/**{sample}.bench**"

  shell: "prinseq++ -fastq {input} -out_good {output} **&> {log}**"

*// Nextflow example*

process prinseq {

  tag "${fq.baseName}"

  input: path(fq)

  output: path(’*.fq’)

  "echo prinseq++ -fastq $fq -out_good ${fq.baseName}.trimmed.fq"

}

*// Failed job’s directory and stdout & stderr will be printed to*

*// the screen and also available in job’s work directory as*

*//*.*command*.*out and*.*command*.*err files*, *respectively*.

*# Ensure benchmarking is recorded in report*.*html & trace*.*txt*

nextflow run <pipeline name> **-with-report -with-trace**

## Rule 2: Utilise flexible configuration stacking for parsing configuration settings

Workflow pipelines often require many different configuration settings. The user may be allowed to tweak some settings, they might *have* to supply others, and there may be settings that the user should not touch. For workflow managers, there are usually several different ways of supplying configurations that can yield a great deal of control when building applications. For convenience, the launcher should accept any settings that the user *must* supply for each run via command line arguments. This can be saved to a configuration file and supplied to the workflow manager on the command line. For optionally customisable settings, the simplest implementation is to copy a default configuration file to the working directory for the user to modify if they wish. In our examples, we copy the default configuration file to the working directory, which the user can tweak. The launcher then updates this file with the command line arguments before supplying it to the workflow manager. The primary benefit for the end user is that their run-time configuration is stored in a directory with their data and is also in a file that can be placed under version control, increasing the reproducibility of the analysis. While user-customised configurations can be passed via the command line, immutable settings should be read directly by the workflow script (this is possible in both Snakemake and Nextflow). This prevents the user from adjusting things that should be left alone but lets the developer keep configuration in a separate file rather than hardcoding these settings.

*# user-customisable settings (config in working directory)*

input: myReads.fastq

output: myTrimmedReads.fastq

prinseqParams: "-min_len 90 -min_qual_mean 25 -ns_max_n 1”

*# immutable settings (config in installation directory)*

databaseDownload:

  mirror:

  "ftp.ncbi.nlm.nih.gov"

  files:

  - blast/demo/MH168512.fsa

  - blast/demo/Plasmids_562.fsa

## Rule 3: Allow flexible execution of workflows

It is quite common for a bioinformatics tool to come with utility scripts for things like installing databases, preparing input files, etc. For these utility scripts, we suggest creating separate workflow scripts that are launched using positional bareword argument subcommands.

% my_tool run…

% my_tool install…

For some workflow managers, it is possible to define individual stages within a workflow that can be run separately. For instance, if the tool performs both preprocessing and assembly, a user might only want to run the preprocessing steps. If possible, you should allow users to define these specific stages to run. In our example, we implement this functionality in Snakemake as additional bareword arguments, and in Nextflow by using the -entry flag.

*# Run preprocessing only (Snakemake)*

% my_tool run… preprocessing

*# Snakefile example*

rule all:

  input: preprocessing_targets, assembly_targets

rule preprocessing:

  input: preprocessing_targets

rule assembly:

  input: assembly_targets

*# Run preprocessing only (Nextflow)*

% my_tool run… -entry preprocessing_workflow

*// Nextflow example*

*// preprocessing step(s) only*

workflow preprocessing_workflow {

  preprocess()

}

*// default workflow*

workflow {

  preprocess | assembly

}

## Rule 4: Embrace the real-time feedback that workflow managers provide

Command line tools that provide meaningful real-time feedback on the progress of a run gives the user confidence that a run is progressing correctly or allows them to intervene if it has stalled or is not running as expected. For this reason, black box applications that do not print anything to the terminal can be frustrating for users. Conversely, some users might prefer minimal terminal messages in which case an option to reduce terminal output would also be beneficial, for instance, via the “quiet” flags that are available for both Snakemake and Nextflow.

Displaying the workflow command and runtime settings tells the user exactly what their system will be doing. For the configuration, a function in either the launcher or the Workflow script can print the configuration settings to the terminal. The runtime configuration, including all file locations, software versions, etc., should also be included in the log to create a record of the data when the analysis was performed. This will also assist with subsequent debugging of any issues that arise.

% my_tool run

  Runtime config settings:

  input: /path/to/infile

  output: my_tool_output/

  Snakemake command:

  Snakemake -j 8 --configfile my_tool_output/config.yaml \

  -s /path/to/my_tool/Snakefile

## Rule 5: Ship software using a package manager to simplify installation

Manually installing software is an unnecessary hurdle for users. Package managers such as Conda and the package installer for Python (pip) will automatically download and install software and represent the easiest avenue for accessing command line applications. Package managers are also an excellent way to help make a tool workflow ready [27], allowing them to be used like any other tool. Conda and pip are two popular cross-platform package managers used in bioinformatics for command line applications. Additional advantages are the ability to install software without root permissions, specify a precise version of the software to use, and create isolated software environments.

Most command line bioinformatics applications are already available to install with Conda from the Bioconda [29] channel and often support both Linux and Mac OSX operating systems. Conda also has the advantage of being a language-agnostic package manager. Python-based tools are usually submitted to the Python Package Index, PyPI, for installation with pip; however, the command line interface can be written in any language and submitted to the package manager for that language (e.g., RubyGem for Ruby, Cargo for Rust, CPAN for Perl).

For workflows-as-applications, submitting to a package manager is especially important as they may require multiple dependencies such as the workflow manager, a command line interface library, and a library to read and write the configuration files. Cross-platform support is also very beneficial but requires careful evaluation across the supported platforms and operating systems. There are many guides available for adding a tool to a package manager repository, and tutorials are usually available in official documentation such as for Python (packaging.python.org) and Conda (docs.conda.io/). For convenience, our Python-based templates generate files to facilitate building and submitting packages to both PyPI and Bioconda.

## Rule 6: Isolate environments and containers for individual steps

Workflows can have many dependencies, and many dependencies increase the chances of conflicts between them. To minimise conflicts and ease reproducibility and maintenance, workflow managers can generate isolated Conda environments or Singularity containers for individual tools and steps, rather than having them already installed. An added advantage of this is that adding the tool to the package manager is easier as it greatly reduces the number of dependencies that must be shipped with the tool. This feature is easy to implement in both Snakemake and Nextflow. In our templates, we include command line options for utilising Conda and some sensible defaults for the Conda settings.

*# Snakefile example*

rule prinseq:

  conda: "../envs/prinseqpp.yaml"

*#* prinseq.*yaml*

name: prinseq

channels:

  - conda-forge

  - bioconda

  - defaults

dependencies:

  - prinseq-plus-plus=1.2.4

*// Nextflow example*

process prinseq {

  conda "bioconda::prinseq-plus-plus=1.2.4"

}

## Rule 7: Clearly identify software environment and database locations

When creating software environments, many workflow managers will save these within subfolders in the working directory. This facilitates reproducibility by keeping everything within a single subdirectory. As a result, every new analysis will generate a whole new set of environment files, which can be wasteful, especially if there are limits on the number of files and folders that can be created on a HPC cluster. Likewise, users may want to specify the installation locations, especially if databases or environments will take up a considerable amount of disk space. Having centralised locations for your environments and databases and allowing these locations to be customised by the user can alleviate this issue. Caution is advised, however, as specifying a location outside of the working or installation directories may have unforeseen consequences, such as files being moved or deleted. As such, many users will prefer to keep environments and databases in the working directory. In our templates, we use the installation directory of the command line tool as the default for both conda environments and databases as this represents the safest centralised location for these files, but users can specify the working directory if they prefer.

## Rule 8: Include a simple test dataset

After installing a new command line tool, users will want a way to quickly and easily verify that a command line tool is working correctly. Often, developers will have a tiny dataset on hand that was used for testing during development. This can be added as an inbuilt test. Adding a function to the launcher can be something as simple as having a “test” argument that updates the input files with the test dataset and then launches the workflow manager like normal. You can even run an md5 checksum on the output files to confirm that the expected results are generated. Another benefit is that this test dataset example can be combined with information to illustrate what the user can expect from the pipeline’s output. While this is not a replacement for unit testing, it is a useful feature to quickly check for issues during development, and users will be grateful for a quick and easy way to test their installations.

% my_tool test

  Running the test dataset

## Rule 9: Follow conventions for HPC cluster support with profiles

Many analyses require lots of computation, which means HPC clusters and schedulers. Workflow managers can submit jobs to a scheduler, usually via a workflow *profile*. A profile differs from a configuration file in that the latter contains settings used in the analysis, whereas a profile contains workflow manager settings. Profiles are a convenient way of running workflow managers on an HPC scheduler. You can also use profiles to specify a set of sensible defaults for the workflow manager, rather than passing them as command line arguments. While profiles can be difficult to set up, there are official examples and templates for running workflow managers across different schedulers (Snakemake: github.com/snakemake-profiles/doc; Nextflow: nf-co.re/docs/usage/tutorials/step_by_step_institutional_profile). The profiles map the resource declarations—such as CPUs, runtime, and memory—in the workflow scripts with the required settings for the HPC’s scheduler. As such, you should ensure your pipeline is compatible with any official or example profiles by using conventional names for declaring resources. You then only need to have your tool optionally accept a declaration for a profile to allow support for both running locally and on an HPC cluster.

*# run locally*

% my_tool run --threads 8…

*# run on a cluster*

% my_tool run --profile slurm…

## Rule 10: Encourage interaction with the workflow manager

You should set some sensible default command line arguments for the workflow manager to make running your tool as easy as possible for all users. However, more experienced users may want to pass their own command line options to the workflow manager, and you should let them do so. In our examples, the launcher achieves this by simply forwarding any unrecognised options to Snakemake or Nextflow. This includes bareword arguments that can be used to specify alternative output files to run specific parts of a workflow (see Rule 3) and is very easy to implement with Python’s Click command line interface. If you are using argparse, you can instead explicitly forward workflow arguments. Snakemake and Nextflow have many command line options and users should be encouraged to investigate these options for their situations.

*# implicitly pass workflow commands (Python Click)*

% my_tool run… --**dry-run**

  Snakemake command:

  snakemake… --**dry-run**

*# explicitly pass workflow commands (Python argparse)*

% my_tool run… –**-next-arg=-with-docker**

  Nextflow command:

  nextflow run… **-with-docker**

## Conclusions

Building workflows-as-applications is beneficial to both developers and end users. These ten simple rules will help address many problems encountered when developing command line tools that are built upon workflow managers and will create a better user experience and a better developer–user relationship. The templates will save even more time in this process as well as offering complete working examples of these ten simple rules for both Snakemake and Nextflow.
